# The role of melatonin in affecting cognitive dysfunction in acute sleep deprivation mice through the nuclear factor kappaB pathway and oxidative stress

**DOI:** 10.1515/tnsci-2025-0379

**Published:** 2025-10-07

**Authors:** Wenting Li, Quan Chen, Haipeng Fu

**Affiliations:** Anesthesiology, The First Clinical School of Medicine, Jinzhou Medical University, No. 40, Section 3, Songpo Road, Linghe District, Jinzhou, 121001, China; Department of Anesthesiology, The First Affiliated Hospital of Jinzhou Medical University, No. 2, Section 5, People Street, Jinzhou, 121001, China

**Keywords:** acute sleep deprivation, cognitive dysfunction, melatonin, oxidative stress, nuclear factor kappaB, postsynaptic density protein 95

## Abstract

**Objective:**

Acute sleep deprivation (ASD) is prevalent in contemporary society. This study explored the mechanism of melatonin affecting cognitive dysfunction (CD) in ASD mice through the nuclear factor kappaB (NF-κB) pathway and oxidative stress.

**Methods:**

The ASD mouse model was established and treated with low-dose and high-dose melatonin, a NF-κB inhibitor PDTC, or lipopolysaccharide (LPS), with their spatial memory, spontaneous activity, and anxiety assessed. Hippocampal morphology and neuronal status were observed via HE and Nissl staining. Superoxide dismutase (SOD) activity and levels of hippocampal CA1 region postsynaptic density protein 95 (PSD95), phosphorylated (p)-p65, and p-IκB proteins; acetylcholinesterase (AChE), acetylcholine (ACh), malondialdehyde (MDA), and reactive oxygen species (ROS); and IL-4, IL-10, tumor necrosis factor [TNF]-α, and IL-1β levels were determined by western blot and ELISA kits.

**Results:**

ASD mice exhibited reduced learning and memory abilities and spontaneous activities, loosely-arranged cells in the hippocampal CA1 region, unclear cell body boundaries, enlarged gaps, severe neuronal damage, and reduced PSD95 protein level. There were increases in AChE, p-p65, p-IκB, TNF-α, IL-1β, MDA, and ROS levels, decrements in ACh, IL-4, and IL-10 levels and SOD activity in the hippocampal CA1 region of ASD mice. Melatonin or PDTC inhibited the NF-κB pathway, down-regulated TNF-α, IL-1β, MDA, and ROS and up-regulated IL-4 and IL-10 and SOD activity in the hippocampal CA1 region of ASD mice, and improved the learning and memory abilities. LPS-induced NF-κB pathway activation partially averted melatonin’s beneficial effects on ASD mice.

**Conclusion:**

Melatonin ameliorated ASD-induced CD in mice by modulating the NF-κB pathway and oxidative stress.

## 1 Introduction

Sleep is a fundamental requirement for all higher life forms, including humans [1]. Sleep deprivation (SD) is known as a shortened sleep time that fails to meet the physiological requirements due to various factors which leads to a range of functional disorders, including metabolic, cardiovascular, endocrine, and nervous system diseases [2]. Inadequate sleep contributes to a general decrease in response speed and an increase in variability in performance, especially in basic measures of alertness, vigilance, and attention [3]. The hippocampus, a crucial component of the medial temporal lobe, is significantly implicated in the conversion and orientation of memory storage, and SD has been found to lead to a reduction in the quantity of neurons and dendritic length within the hippocampal CA1 region [4]. Additionally, SD activates microglia and astrocytes, bringing about elevated levels of pro-inflammatory factors, leading to neuroinflammation, which in turn fosters oxidative stress, culminating in brain damage [5,6]. Despite the increasing number of studies on SD in recent years, the underlying mechanism of cognitive dysfunction (CD) remains incompletely understood.

Oxidative stress is a state caused by an imbalance between reactive oxygen species (ROS) and the antioxidant defense system, and is associated with various diseases such as diabetes, neurodegenerative diseases, and cardiovascular diseases [7]. SD can cause activation of astrocytes and microglia to facilitate the secretion of pro-inflammatory factors and trigger neuroinflammation, which in turn promotes oxidative stress, ultimately resulting in brain injury, one manifestation of which is CD [5,6]. A study conducted on both animal models and human subjects with obstructive sleep apnea syndrome has demonstrated that SD leads to increased oxidative stress [8]. This is primarily due to the fact that SD causes an imbalance in brain redox status, substantial increases in oxidative damage markers, such as malondialdehyde (MDA), and a decrease in superoxide dismutase (SOD) in cognitive-critical regions like the hippocampus [9–11]. A prior study has elucidated the activation of oxidative stress and integrated stress response pathways in gamma-aminobutyric acid-ergic neurons due to SD [12]. It has been documented that up-regulation of the activity of antioxidant enzymes in the brain can reduce oxidative stress, thereby improving memory and learning impairments caused by SD [13].

The nuclear factor kappaB (NF-κB), which plays a crucial role in inflammation, cell adhesion, growth signals, host immune response, cell differentiation, cell proliferation, and apoptosis defense, is identified as a dimeric transcription factor [14]. The NF-κB pathway pitches in SD-induced neuroinflammation [15]. The activation of NF-κB up-regulates pro-inflammatory cytokines (such as interleukin [IL]-4, IL-10, tumor necrosis factor [TNF]-α), leading to the exacerbation of oxidative stress and inflammatory responses [16]. Furthermore, it has been documented that SD rats exhibit activated NF-κB pathway [17]. Various active components found in traditional Chinese medicine have been shown to mitigate hippocampal inflammation by suppressing the NF-κB pathway and then improving memory and learning impairment in SD rats [18,19]. However, the mechanism of NF-κB in acute sleep deprivation (ASD)-induced CD in mice remains elusive.

Melatonin, a methoxyindole, is primarily synthesized and secreted by the pineal gland during the night under typical light/dark cycles [20]. Melatonin has the ability to traverse the blood–brain barrier and gain access to various brain regions where it can mitigate neurological inflammation and oxidative damage [21]. Gut microbe-derived metabolites modulate the neuroprotective properties of melatonin in cognitive impairment induced by SD [22]. Melatonin mitigates CD and hippocampal ferroptosis induced by acute SD by interacting with the MT2 receptor to initiate the ERK/Nrf2 pathways [23]. Furthermore, melatonin augments NF-κB deacetylation, leading to a subsequent decrease in the inflammation [24]. However, the mechanism of action by which melatonin affects CD in ASD mice through the NF-κB pathway and oxidative stress has been minimally addressed in literature. Based on this context, this study probed the mechanism of melatonin affecting CD in ASD mice through the NF-κB pathway and oxidative stress to offer a theoretical basis for treating CD resulting from ASD.

## 2 Materials and methods

### 2.1 Experimental animals

A total of 60 male C57BL/6J mice (20 months old, body weight 30–35 g, Avanc Pharmaceutical Co., Ltd, SYXK (Liaoning) 2022-0006, Jinzhou, Liaoning, China) were maintained under standard environment in a 12 h light–dark cycle, at 21 ± 1°C, with 50 ± 10% relative humidity and *ad libitum* access to water and food. The subsequent experiments were carried out after 7 days of acclimatization.

### 2.2 Experimental design

Establishment of ASD mouse model was realized using the multi-platform water environment method [25–27]. Reportedly, female C57BL/6J mice have shorter total sleep time and exhibit less non-rapid eye movement sleep and more wakefulness during the dark phase (active phase) [28]. These differences may be related to lower physiological sleep requirements in females [28]. Therefore, male mice have been used in multiple SD models [22,29–31]. Additionally, considering the effect of estrogen on the NF-κB pathway [32–36], male C57BL/6J mice were selected for the establishment of the SD animal model.

The ASD mouse model was established using the multi-platform water environment method based on previous studies [25–27]. The ASD device was a water sink (120 cm × 60 cm × 40 cm). Within the sink, there were 20 wooden cylinders with a diameter of 3.5 cm, arranged in four columns (five cylinders in each column), with a 5 cm spacing between cylinders. The sink was filled with water at 24 ± 1°C until the water level reached 2 cm below the flat of the cylinders. A grid was placed over the top of the sink, through which the feed and water bottles were suspended above the top of the sink. Mice were permitted unrestricted access to food and water on the platforms and were able to traverse between them by jumping, but unable to rest across two platforms. When the mice entered the rapid eye movement sleep, their muscle tension decreased. The mice then fell into the water and woke up and climbed back onto the platform. The above process ensured that the mice were awake to achieve the effect of ASD. The experiment was commenced at 8 a.m. on the initial day, with 18 h ASD conducted per day for 3 consecutive days.

The mice were placed on a wide-platform sink with a cylindrical diameter of 12 cm to enable them to rest on the cylindrical surface without the risk of falling into the water, which was designated as the control group. The water in the sink was refreshed daily to maintain a hygienic experimental setting.

### 2.3 Animal grouping and treatment

The mice were randomly arranged into the following six groups (12 mice/group): (1) the control group (control mice); (2) the SD group (ASD mice); (3) the SD + L-MEL group (ASD mice were injected with low-dose melatonin [20 mg/kg, HY-B0075, MedChemExpress, Monmouth Junction, NJ, USA] intraperitoneally [37]); (4) the SD + H-MEL group (ASD mice were injected with high dose melatonin [40 mg/kg, HY-B0075, MedChemExpress] intraperitoneally [38]); (5) the SD + PDTC group (ASD mice were intraperitoneally injected with 200 mg/kg of a NF-κB inhibitor PDTC [HY-18738, MedChemExpress] dissolved in saline [39]); (6) the SD + H-MEL + LPS group (mice received intraperitoneal injections of 40 mg/kg melatonin and 750 μg/kg lipopolysaccharide [LPS; HY-D1056, MedChemExpress] [40]). Three injections of the latter three group were conducted 1 h before ASD (at 7 a.m. daily). Both the control and ASD mice received intraperitoneal injections of equal amounts of saline at the corresponding times.

After ASD, each group underwent Morris water maze (MWM) test, open field test, and elevated plus maze test. All mice were sacrificed by intraperitoneal injection of 3% sodium pentobarbital (150 mg/kg; P3761, Sigma-Aldrich, St Louis, MO, USA) immediately after the behavioral experiments. Hippocampal CA1 region tissues of six mice were made into tissue homogenates for enzyme-linked immunosorbent assay (ELISA) and western blot analysis, while those from the remaining six mice were utilized for hematoxylin and eosin (HE) and Nissl staining. The specific experimental flowcharts are shown in Supplementary figure 1.

### 2.4 MWM test

MWM test was used to evaluate the spatial learning and memory abilities of animals [25,41]. A circular stainless steel pool, measuring 120 cm in diameter and 60 cm in height, was partitioned into four quadrants of equal size. In the center of the target quadrant, a circular hidden platform with a diameter of 10 cm was positioned approximately 1–2 cm below the water surface. The water temperature was maintained within the range of 23–25°C, and a MWM video analysis system (SA201, SansBio, Nanjing, Jiangsu, China) was installed above the pool. The experiment comprised a training phase lasting for 5 days and a testing phase lasting for 1 day. One day before training, each cohort of mice was, respectively, placed in the pool without a platform for 60 s to allow them to acclimate to the environment. At 9:00 each day during the training session, the mice were sequentially introduced into the pool facing walls from the four quadrant and compelled to submerge themselves to locate the target platform. The escape latency was measured using an image-self monitoring system, which documented the time taken by the mice to climb up from the water to reach the target platform. The mice were immersed in water for 60 s during each trial and kept on the target platform for 10 s. In cases where the mice were unable to reach the target platform within the allotted 60 s, they were assisted in climbing to the platform, and the time was recorded as 60 s. The mice underwent four training sessions per day at an interval of 60 min, with the escape latency recorded. The water maze was cleaned and refreshed after each day’s training to eliminate the influence of the environment on the experiment. On the sixth day, the underwater platform was removed, and mice were released into the water from the opposite quadrant of the original target platform. The time and number of times for each mouse first crossed the original platform and time spent in the original target platform quadrant within 180 s was recorded.

### 2.5 Open field test

As described in previous research [42–45], the open field experiment is a standardized behavioral assay widely used to evaluate cognitive function, anxiety-like behaviors, and locomotor activity in rodents. The open field experimental box was a square open box measuring 50 cm × 50 cm × 40 cm. Spontaneous activity and mental anxiety could be assessed by examining the movement trajectory and movement area of animals within the box. At the beginning of the experiment, the experimenter quickly placed the mice in the central area of the open field experiment box with their back facing the experimenter, and then immediately left. Subsequently, the mice were granted unrestricted movement within the box for 10 min. The movement trajectories and distances covered by the animals in the final 4 min of the experimental session within the box were documented utilizing an open-field test system (SA215S, SansBio). After the experiment of each mouse, the box underwent a complete cleaning to prevent any residual excreta or odor that could potentially impact the outcomes of the next mouse.

### 2.6 Elevated plus maze test

The elevated plus maze consisted of two opposing open arms, two opposing enclosed arms, and a central platform [46]. Experimental mice were placed at the maze center for 5 min, with their time spent in the open and enclosed arms recorded.

### 2.7 HE staining

The hippocampal CA1 region of mice was fixed with 4% paraformaldehyde (PFA; AWI0056b, Abiowell, Changsha, Hunan, China) for 24 h and then subjected to routine dehydration using an automatic tissue dehydrator (HD-300B, Huida, Xiaogan, Hubei, China), embedding using a paraffin embedding machine (HD-310B, Huida), and then sectioning at 4 μm thickness using a rotary sectioner (HM-325, Thermo Fisher, San Jose, CA, USA). Subsequently, the sections were dewaxed, hydrated, and washed with phosphate buffer saline (PBS), followed by HE staining with HE staining solution (AWI0020b; Abiowell). The sections were later dehydrated using gradient ethanol, cleared in xylene, sealed with neutral gum, and finally photographed under a microscope.

### 2.8 Nissl staining

The hippocampal CA1 region of mice was fixed with 4% PFA for 24 h, routinely dehydrated and embedded, followed by cutting into 5 μm thick sections. Then, the sections were dewaxed and hydrated. After washing with PBS, sections were immersed in Nissl staining solution (AWI0502a; Abiowell), incubated at 56°C for 1 h, rinsed in distilled water and differentiated in Nissl differentiation solution for 2 min. Afterward, sections were dehydrated by gradient ethanol, cleared in xylene, and sealed with neutral gum prior to observation and photographing using a microscope.

### 2.9 ELISA

ELISA kits were used to determine levels of IL-4 (Mouse IL-4 ELISA Kit, ml063156, Enzyme-linked Biotechnology, Shanghai, China), IL-10 (Mouse IL-10 ELISA Kit, ml037873, Enzyme-linked Biotechnology), TNF-α (Mouse TNF-α ELISA Kit, ml002095, Enzyme-linked Biotechnology), IL-1β (Mouse IL-1β ELISA Kit, ml301814, Enzyme-linked Biotechnology), acetylcholine (ACh) (mouse ACh ELISA kit, E-EL-0081, Elabscience, Wuhan, Hubei. China), acetylcholinesterase (AChE) (mouse AChE ELISA kit, E-EL-M2637, Elabscience), MDA (Mouse MDA ELISA Kit, ml-E5148, Enzyme-linked Biotechnology), ROS (Mouse ROS ELISA Kit, ml-E5148, Enzyme-linked Biotechnology), and SOD (Mouse SOD ELISA Kit, ml643059, Enzyme-linked Biotechnology) levels in the hippocampal CA1 region of mice were determined using ELISA kits. Specific procedures were performed in the light of kit instructions.

### 2.10 Western blot

The hippocampal CA1 region of mice was collected. Total protein of tissues was extracted using the total protein extraction kit (P0033, Beyotime, Shanghai, China), and protein concentration was measured using the bicinchoninic acid protein detection kit (P0009, Beyotime) according to the instructions provided by the supplier. Protein samples (20 µg/well) were electrophoresed on a 12% sodium dodecyl sulfate polyacrylamide gel electrophoresis gel, transferred onto a polyvinylidene difluoride membrane and then blocked with 5% skim milk for 1 h. Thereafter, membranes were cultivated with primary antibodies against postsynaptic density protein 95 (PSD95) (1:2,000, ab238135; Abcam, Cambridge, UK), p-p65 (1:2,000, ab239882; Abcam), p65 (0.5 µg/mL, ab16502; Abcam), phosphorylated (p)-IκB (1:10,000, ab133462; Abcam), IκB (1:10,000, ab32518; Abcam), and β-actin (1:1,000, ab8227; Abcam) overnight at 4°C. After 3 tris-buffered saline with Tween 20 washes, membranes were fostered with horseradish peroxidase-labeled goat anti-rabbit secondary antibody immunoglobulin G (1:5,000, ab6721; Abcam) at room temperature for 1 h. Subsequently, membranes were developed with enhanced chemiluminescence working solution (Omni-ECL™ Enhanced Chemiluminescence Detection Kit, SQ201, Epizyme, Shanghai, China). The grayscale was analyzed using the ImageJ software (RRID: SCR_003070, National Institutes of Health, Bethesda, MD, USA).

### 2.11 Statistical analysis

All data were statistically analyzed and graphed using the GraphPad Prism 8.0.1 software (GraphPad Software, San Diego, CA, USA). Data were presented as mean ± standard deviation, with *t-*test conducted for comparisons between two groups, and one-way analysis of variance (ANOVA) for comparisons among multiple groups, followed by Tukey’s test. *P* was a two-sided test. Escape latency in the MWM test was analyzed using two-way ANOVA, with the Šídák’s multiple comparison test used for *post hoc* analyses. The level of significance was *P* < 0.05. All multiple comparisons were subjected to false discovery rate (FDR) correction using the Benjamini–Hochberg method (*q* = 0.05). Corrected *P*-values were denoted as *P*_adj_, while uncorrected raw *P*-values were labeled as *P*. FDR correction (*q* = 0.05) was applied to all multiple comparisons within each analytical category (inflammatory cytokines, oxidative stress markers). However, primary outcome analyses (MWM and OFT) were not subjected to multiplicity correction as they represented our pre-specified primary endpoints; comparisons across different categories (western blot) were not corrected as they addressed distinct biological issues; histological results (HE and Nissl staining) were descriptive or semi-quantitative to support the quantitative findings, and no FDR correction was applied.


**Ethical approval:** The research related to animals’ use has been complied with all the relevant national regulations and institutional policies for the care and use of animals. All animal experiments in this study were reviewed and ratified by the Animal Ethics Committee of Jinzhou Medical University, approval number 2023061301. All efforts were made to minimize the animals’ suffering.

## 3 Results

### 3.1 Melatonin improved CD in ASD mice

**Figure 1:**
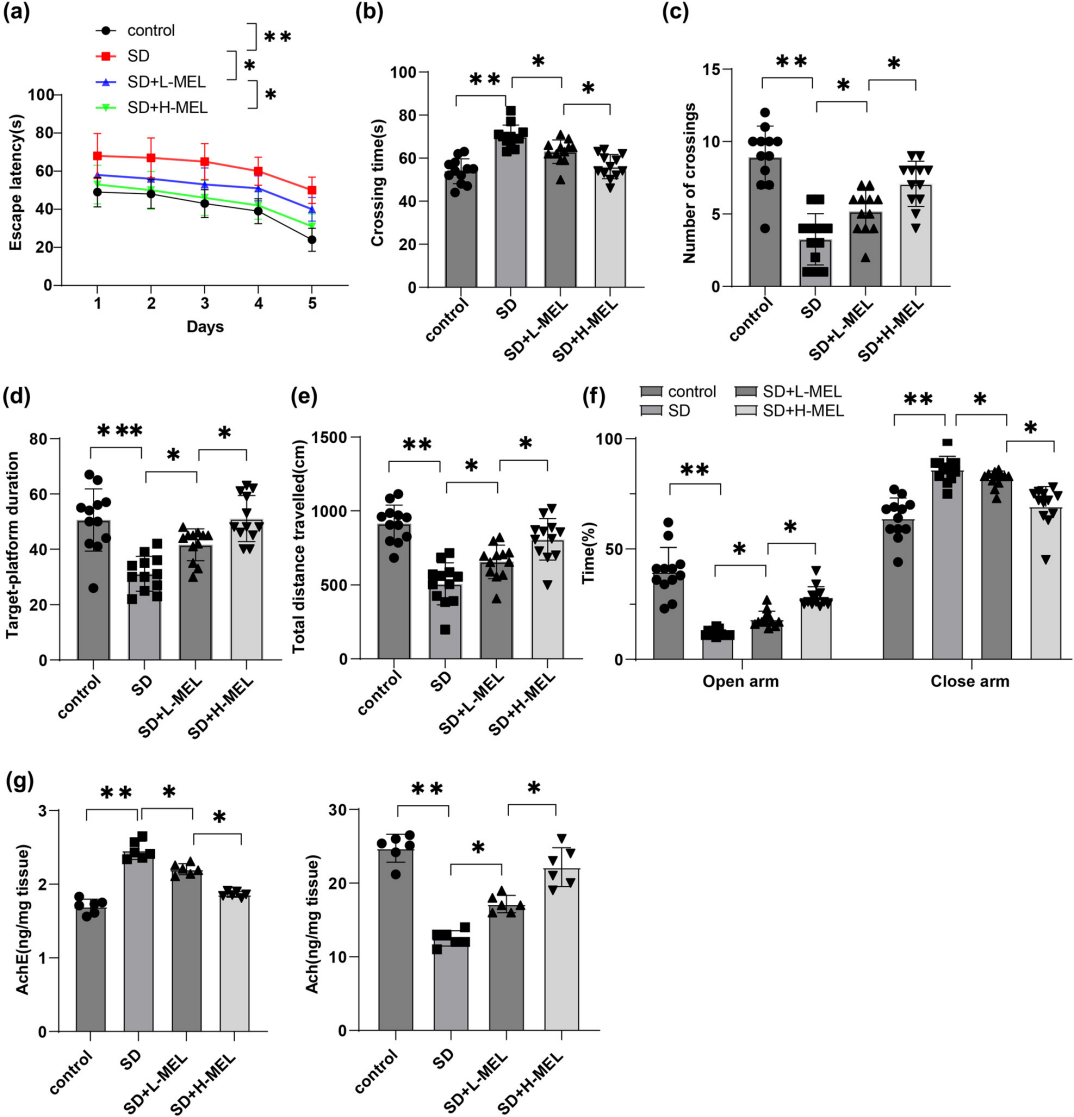
Melatonin improved CD in male C57BL/6J ASD mice. MWM test was conducted to detect (a) escape latency, (b) time needed for first crossing, (c) number of crossings through the target platform zone, and (d) time spent in the target platform quadrant within 180 s of mice (*n* = 12); (e) open field experiment was performed to assess mouse spontaneous activity (*n* = 12); (f) elevated plus maze test was conducted to assess time spent in open and enclosed arms (*n* = 12); (g) ELISA was used to measure AChE activity and ACh levels in the hippocampal CA1 region of SD mice (*n* = 6). Data in panel (c) failed the Shapiro–Wilk test for normality and the Mann–Whitney *U*-test was applied for inter-group comparisons. All other data were normally distributed, with the results presented as mean ± standard deviation. (a) Escape latency in the MWM test was analyzed using two-way ANOVA, with the Šídák’s multiple comparison test used for *post hoc* analyses; (b)–(g) one-way ANOVA was applied for comparisons among multiple groups, and Tukey’s multiple comparison test for *post hoc* analysis. *P < 0.05, **P < 0.01.

Electroencephalography (EEG) results confirmed that ASD mice remained awake for an average of approximately 95% of the time during the 18 h ASD period over 3 consecutive days (Supplementary file 1) (*P* < 0.001). As reflected by MWM experiment, ASD mice exhibited a remarkable increment in escape latency, an increase in the time for their first crossing, a decrease in the number of crossing through the target platform zone, and a reduction in time spent in the target platform quadrant (all *P* < 0.01) (Figure 1a–d). The performance of melatonin-intervened mice was better than that of the SD group, as evidenced by reduced escape latency, shortened time for their first crossing, increased crossing number through the target platform zone, and reduced time spent in the target platform quadrant, with the changes dose-dependent on melatonin (all *P* < 0.05) (Figure 1a–d). As demonstrated by open field experiment, the total distance the mice travelled was reduced in the SD group versus the control group, while after melatonin treatment, the total distance the ASD mice traveled was increased, with the changes dependent on the dosage of melatonin (all *P* < 0.05) (Figure 1e). In the elevated plus maze test, mice in the SD group spent less time in the open arms and more time in the enclosed arms compared to those in the control group. Following melatonin treatment, ASD mice exhibited increased time in the open arms and diminished time in the enclosed arms, with these changes being dose-dependent (Figure 1f, all *P* < 0.05). ACh is a crucial neurotransmitter in the central nervous system. Overactivity of AChE accelerates the degradation of ACh, and decreased ACh levels are associated with CD [47]. Levels of ACh and AChE activity in the hippocampal CA1 region of SD mice were measured by ELISA. Results revealed that compared to the control group, mice in the SD group had increased AChE activity and decreased ACh levels. Melatonin treatment resulted in decreased AChE activity and increased ACh levels in SD mice, with these changes being dose-dependent with melatonin (all *P* < 0.05) (Figure 1g). These results suggested that ASD could cause CD in mice, and melatonin could alleviate ASD-induced CD.

### 3.2 Melatonin attenuated neuroinflammation and oxidative stress in the hippocampal CA1 region of ASD mice

**Figure 2:**
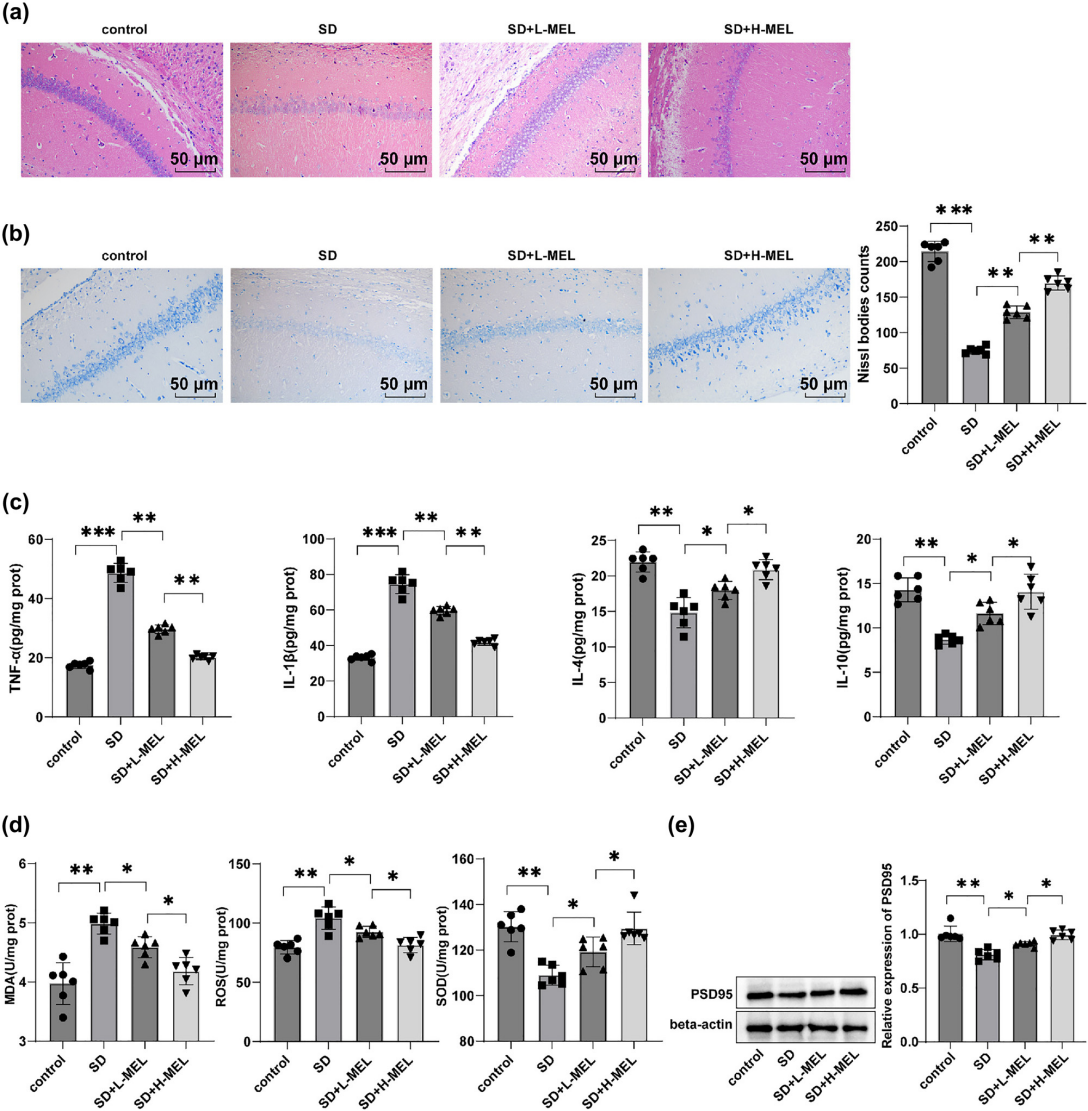
Melatonin mitigated neuroinflammation and oxidative stress in the hippocampal CA1 region of male C57BL/6J ASD mice. (a) HE staining to observe the structure of the hippocampal CA1 region of mice; (b) Nissl staining to observe the neurons of the hippocampal CA1 region of mice; ELISA to determine TNF-α, IL-1β, IL-4, and IL-10, (c) MDA and ROS levels, and SOD activity in the hippocampal CA1 region of mice (d); (e) western blot to measure PSD95 protein expression in the hippocampal CA1 region of mice; *n* = 6. All data were normally distributed as determined by the Shapiro–Wilk test and were presented as mean ± standard deviation. One-way ANOVA was applied for comparisons among multiple groups, and Tukey’s multiple comparison for *post hoc* analysis. Multiple comparisons were subjected to FDR correction using the Benjamini–Hochberg method (*q* = 0.05). Corrected *P*-values were denoted as *P*_adj_, while uncorrected raw *P*-values were labeled as *P*. FDR correction (*q* = 0.05) was applied to all multiple comparisons within each analytical category (inflammatory cytokines, oxidative stress markers). *P < 0.05, **P < 0.01.

HE and Nissl staining were conducted to observe the pathological changes of hippocampal CA1, with results showing that relative to the control group, the cells of the hippocampal CA1 region were loosely arranged, the cell body boundary was unclear, the gap was increased, the neurons were severely damaged, and Nissl bodies were reduced in mice of the SD group (*P* < 0.001). In contrast, melatonin-treated mice displayed dramatically improved hippocampal CA1 region, neatly arranged cells, reduced gaps, alleviated neuronal damage, and increased Nissl bodies (all *P* < 0.01) and the mitigating effects of the SD + H-MEL group were better than those of the SD + L-MEL group (Figure 2a and b). With regard to the ELISA results, the levels of pro-inflammatory factors (TNF-α and IL-1β) were raised and the levels of anti-inflammatory factors (IL-4 and IL-10) in the hippocampal CA1 region were decreased (all *P*_adj_ < 0.01) (Figure 2c), and the levels of oxidative stress-related indicators (MDA and ROS) in the hippocampal CA1 region were hoisted and SOD activity was abated (all *P*_adj_ < 0.01) (Figure 2d) in the SD group compared with the control group. Melatonin treatment reduced TNF-α and IL-1β levels in the hippocampal CA1 region and increased IL-4 and IL-10 levels (all *P*_adj_ < 0.05) (Figure 2c), and reduced MDA and ROS levels in hippocampal CA1 region and enhanced SOD activity, with the changes dose-dependent on melatonin (all *P*_adj_ < 0.05) (Figure 2d). Western blot results revealed that PSD95 protein level in the hippocampal CA1 region of mice in the SD group was lower than the control group (all *P* < 0.01) (Figure 2e), whereas melatonin up-regulated PSD95 protein level (all *P* < 0.05) (Figure 2e). In conclusion, ASD caused neuronal damage, inflammation, and oxidative stress in hippocampal CA1 region of mice, and melatonin could improve these pathological changes.

### 3.3 Melatonin inhibited the activation of the NF-κB pathway

**Figure 3:**
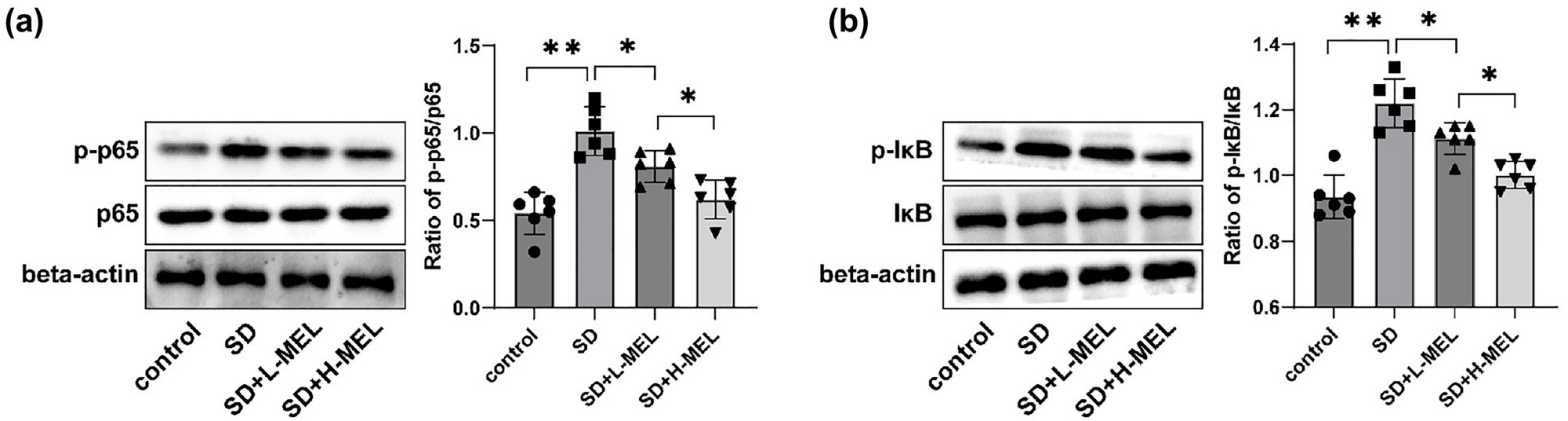
Melatonin repressed the NF-κB pathway activation in male C57BL/6J ASD mice. Western blot was implemented to determine (a) p-p65/p65 ratio and (b) p-IκB/IκB ratio in the hippocampal CA1 region of mice; *n* = 6. All data were normally distributed as determined by the Shapiro–Wilk test and were presented as mean ± standard deviation. Comparisons among multiple groups were analyzed using one-way ANOVA, with Tukey’s multiple comparison test utilized for *post hoc* analysis. *P < 0.05, **P < 0.01.

To further explore the mechanism by which melatonin affected CD in ASD mice, p-p65, p65, p-IκB, and IκB protein levels in the hippocampal CA1 region of mice were assessed by western blot. In comparison to the control group, the hippocampal CA1 region of mice exhibited increased p-p65/p65 and p-IκB/IκB ratios in the SD group (all *P* < 0.01) (Figure 3a and b). Conversely, the p-p65/p65 and p-IκB/IκB ratios were reduced in the SD + L-MEL and SD + H-MEL groups versus the SD group (all *P* < 0.05) (Figure 3a and b). The aforementioned results indicated that the NF-κB pathway in the hippocampal CA1 region of mice was activated, whereas it was inhibited following melatonin treatment.

### 3.4 Inhibition of the NF-κB pathway ameliorated neuroinflammation and oxidative stress in the hippocampal CA1 region of ASD mice

**Figure 4:**
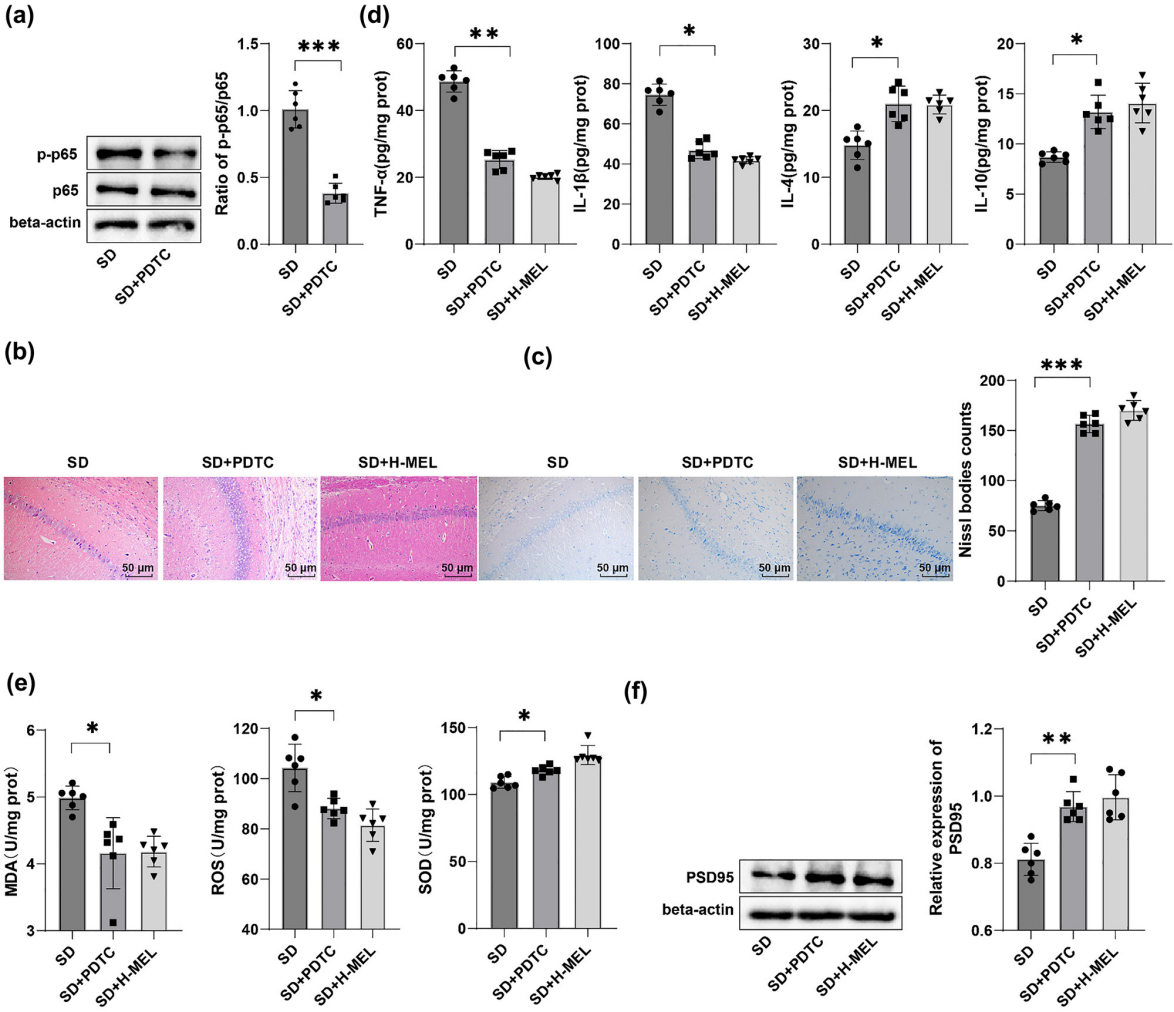
Suppression of the NF-κB pathway improved neuroinflammation and oxidative stress in the hippocampal CA1 region of male C57BL/6J ASD mice. (a/f) Western blot to determine p-p65/p65 ratio and PSD95 protein expression in the hippocampal CA1 region of mice; (b) HE staining to observe the structure of the hippocampal CA1 region of mice; (c) Nissl staining to observe the neuronal status of the hippocampal CA1 region of mice; ELISA to determine (d) TNF-α, IL-1β, IL-4, and IL-10 levels and (e) MDA levels, ROS, and SOD activity in the hippocampal CA1 region; *n* = 6. Data were expressed as mean ± standard deviation. The *t*-test was adopted for inter-group comparisons. Multiple comparisons were subjected to FDR correction using the Benjamini–Hochberg method (*q* = 0.05). Corrected *P*-values were denoted as *P*_adj_, while uncorrected raw *P*-values were labeled as *P*. FDR correction (*q* = 0.05) was applied to all multiple comparisons within each analytical category (inflammatory cytokines, oxidative stress markers). *P < 0.05, **P < 0.01, ***P < 0.001.

Relative to the SD group, the ratio of p-p65/p65 in the hippocampal CA1 region was decreased in the SD + PDTC group (*P* < 0.001) (Figure 4a). The HE and Nissl staining results unveiled a notable improvement in the hippocampal CA1 region of mice in the SD + PDTC group compared to the SD group, as evidenced by well-aligned cells, diminished gaps, reduced neuronal damage, and increased Nissl bodies (*P* < 0.001) (Figure 4b and c). Subsequently, the ELISA results indicated decreases in levels of hippocampal CA1 region TNF-α and IL-1β and increments in IL-4 and IL-10 levels (all *P*_adj_ < 0.05) (Figure 4d), as well as reductions in MDA and ROS levels in the hippocampal CA1 region and an enhancement in SOD activity (all *P*_adj_ < 0.05) (Figure 4e) in the SD + PDTC group relative to the SD group. Furthermore, western blot results demonstrated that PSD95 protein level in the hippocampal CA1 region of the SD + PDTC group was higher than that of the SD group (*P* < 0.01) (Figure 4f). The SD + PDTC group and the SD + H-MEL group exhibited similar therapeutic efficacy in ameliorating neuroinflammation and oxidative stress in SD mice (Figure 4b–f). The aforesaid results unraveled that inhibition of the NF-κB pathway ameliorated neuroinflammation and oxidative stress in the hippocampal CA1 region of ASD mice.

### 3.5 Repression of the NF-κB pathway mitigated CD in ASD mice

**Figure 5:**
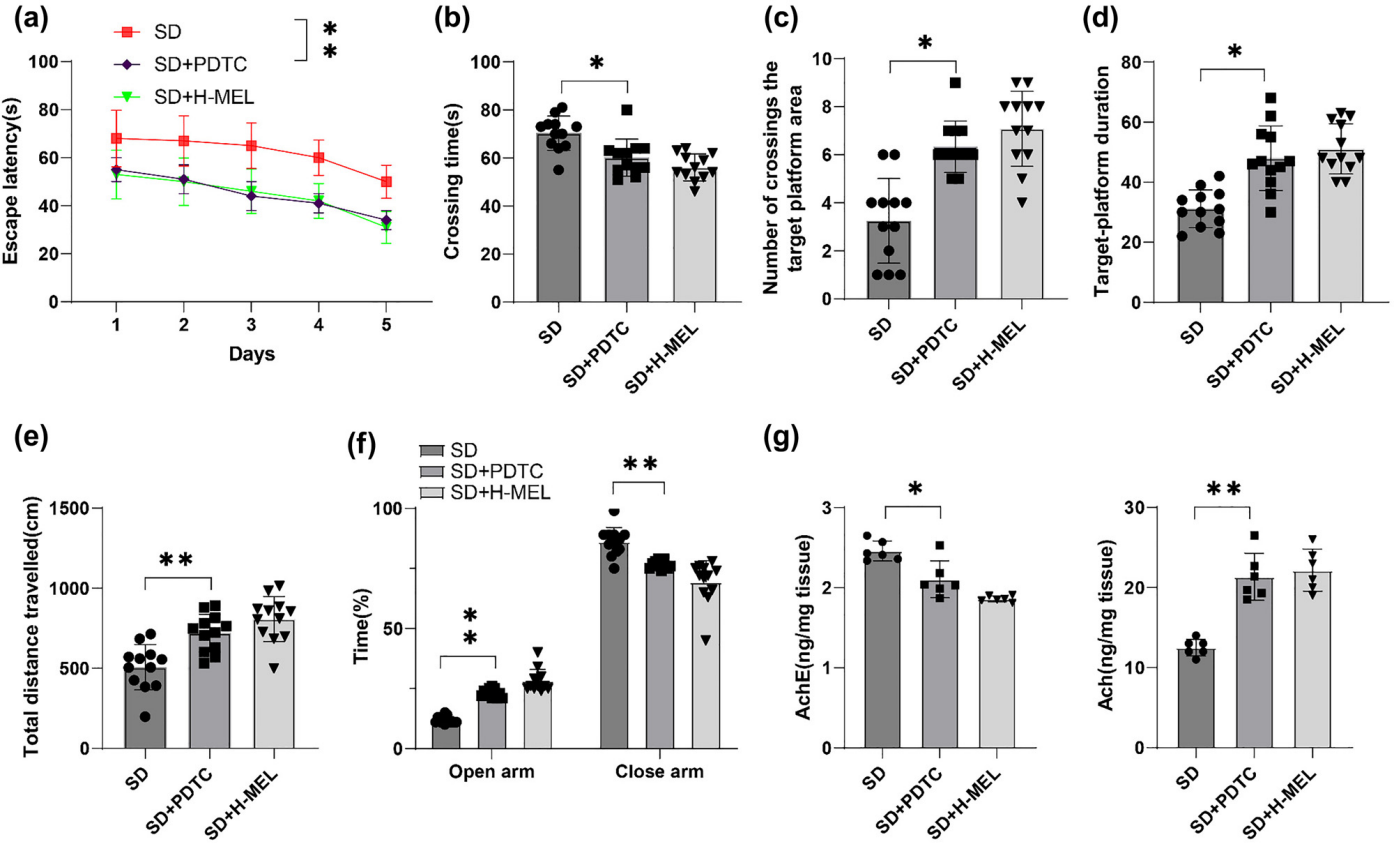
Inhibition of the NF-κB pathway notably attenuated CD in male C57BL/6J ASD mice. MWM test to determine (a) escape latency, (b) first crossing time, (c) number of crossings through the target platform zone, and (d) time spent in the target platform quadrant within 180 s of mice (*n* = 12); (e) open field experiment to assess spontaneous activity of mice (*n* = 12); (f) elevated plus maze test was conducted to assess time spent in open and enclosed arms (*n* = 12); (g) ELISA was used to measure AChE activity and ACh levels in the hippocampal CA1 region of SD mice (*n* = 6). Data were expressed as mean ± standard deviation. (a) Escape latency in the MWM test was analyzed using two-way ANOVA, with the Šídák’s multiple comparison test used for *post hoc* analyses; (b)–(g) the *t* test was utilized for inter-group comparisons. **P < 0.01.

To confirm the impact of the NF-κB pathway on CD in ASD mice, spatial memory and spontaneous activity were assessed by MWM and open field experiments. The results of the MWM experiment indicated that the mice in the SD + PDTC group presented a notable decrease in escape latency, a reduction in the time needed for first crossing, an increase in the number of crossing through the target platform zone, and increased time spent in the target platform quadrant compared to the SD group (*P* < 0.01) (Figure 5a–d). Moreover, open field experiment demonstrated a dramatic enhancement in the total distance traveled in mice of the SD + PDTC group versus the SD group (*P* < 0.01) (Figure 5e). The elevated plus maze test results demonstrated that, compared with the SD group, the SD + PDTC group spent more time in the open arms and less time in the enclosed arms (all *P* < 0.01) (Figure 5f). AChE activity was reduced and ACh levels were elevated in the SD + PDTC group (both *P* < 0.01) (Figure 5g). The SD + PDTC and SD + H-MEL groups had similar effects on alleviating CD in ASD mice (Figure 5a–g). The results manifested that the suppression of the NF-κB pathway notably reduced CD in ASD mice.

### 3.6 Activation of the NF-κB pathway partially abolished the beneficial effects of melatonin on oxidative stress and CD in the hippocampal CA1 region of ASD mice

**Figure 6:**
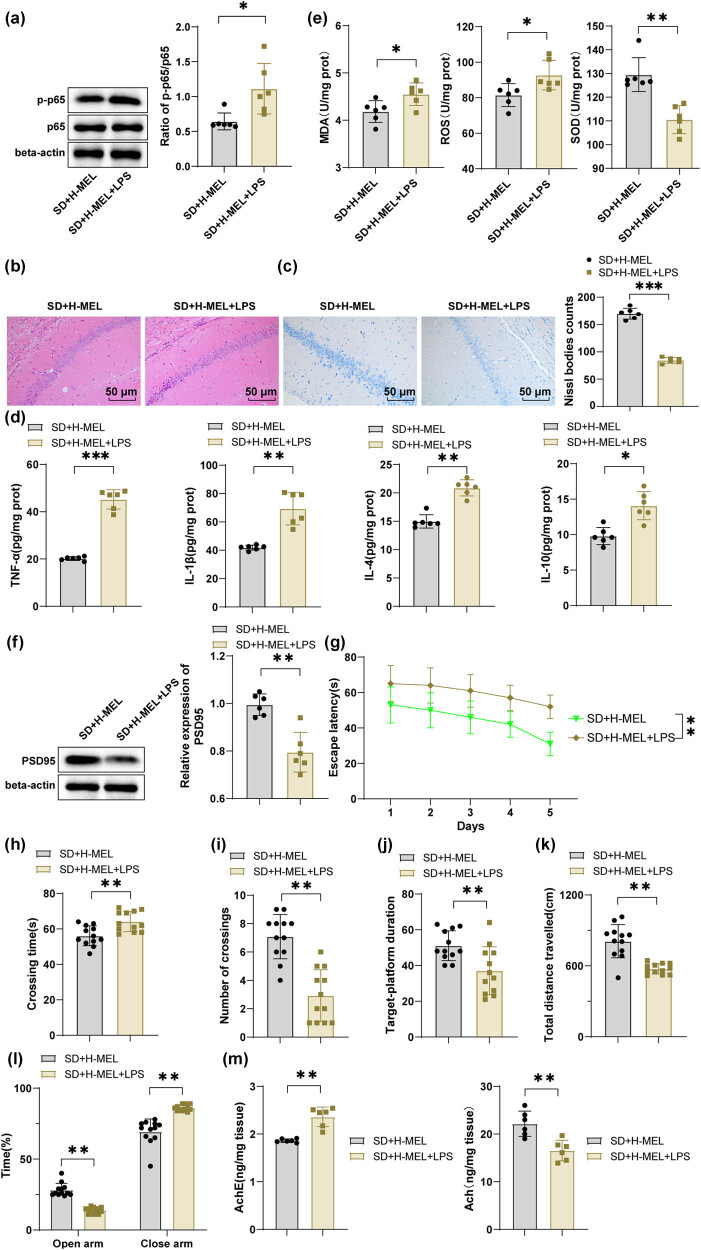
Activation of the NF-κB signaling promoted oxidative stress and CD in the hippocampal CA1 region of male C57BL/6J ASD mice. (a/f) Western blot for determination of p-p65, p65, and PSD95 protein expression levels in the hippocampal CA1 region (*n* = 6); (b) HE staining for observation of the structure of the mouse hippocampal CA1 region (*n* = 6); (c) Nissl staining for assessment of neuronal status in the mouse hippocampal CA1 region (*n* = 6); (d) ELISA for measurement of levels of TNF-α, IL-1β, IL-4, and IL-10 levels in the hippocampal CA1 region of mice (*n* = 6); (e) levels of MDA, ROS, and SOD activity in the mouse hippocampal CA1 region (*n* = 6); (f) western blot for determination of PSD95 protein expression; MWM test to detect (g) escape latency, (h) time needed for first crossing, (i) number of crossings through the target platform zone within 180 s, and (j) time spent in the target platform quadrant within 180 s of mice (*n* = 12); (k) open field experiment to assess mouse spontaneous activity (*n* = 12); (l) elevated plus maze test to quantify time spent in open and enclosed arms (*n* = 12); (m) ELISA was used to measure AChE activity and ACh levels in the hippocampal CA1 region of SD mice (*n* = 6). Data were presented as mean ± standard deviation. (g) Escape latency was analyzed using two-way ANOVA, with the Šídák’s multiple comparison test used for *post hoc* analyses; other panels: inter-group comparisons were analyzed by the *t*-test. Multiple comparisons underwent FDR correction via the Benjamini–Hochberg method (*q* = 0.05). Corrected *P*-values were denoted as *P*_adj_ and uncorrected raw *P*-values as *P*. FDR correction (*q* = 0.05) was applied to all multiple comparisons within each analytical category (inflammatory cytokines, oxidative stress markers). *P < 0.05, **P < 0.01, ***P < 0.001.

Finally, the NF-κB pathway was activated using LPS to further validate the critical role of the NF-κB signaling in ameliorative effects of melatonin on oxidative stress and CD in the hippocampal CA1 region of ASD mice. It was found that compared to the SD + H-MEL group, the mouse hippocampal CA1 region in the SD + H-MEL + LPS group exhibited an increased p-p65/p65 ratio, decreased PSD95 protein expression (both *P* < 0.01) (Figure 6a/f), and reduced Nissl bodies (*P* < 0.001). Additionally, LPS also resulted in aggravated neuronal damage (Figure 6b and c), elevated TNF-α and IL-1β levels, diminished IL-4 and IL-10 levels (all *P*_adj_ < 0.01) (Figure 6d), up-regulated MDA and ROS levels, and weakened SOD activity (all *P*_adj_ < 0.05) (Figure 6e). As reflected by behavioral test results, relative to the SD + H-MEL group, the SD + H-MEL + LPS group had increased escape latency, prolonged first platform crossing time, reduced platform crossings through the target platform zone, declined time spent in the target platform quadrant, diminished total distance traveled in the open field, decreased time in open arms, increased time in enclosed arms, and increased AChE activity and decreased ACh levels in the hippocampal CA1 region (all *P* < 0.01) (Figure 6g–m). These findings indicated that NF-κB pathway activation partially reversed the ameliorative effects of melatonin on oxidative stress and CD in the hippocampal CA1 region of ASD mice.

## 4 Discussion

With the advancement of contemporary society and the acceleration of the pace of life, SD has emerged as a critical issue impacting the work and daily lives of individuals [48]. SD potentially causes CD [49]. The involvement of oxidative stress and inflammation has been suggested in impairments related to SD [6]. NF-κB upregulation stimulates pro-inflammatory cytokine gene expression levels and ultimately results in neuronal damage [17]. Moreover, the preliminary study indicates that melatonin is effective in mitigating SD-caused impairments [23]. Accordingly, the present study revealed that melatonin ameliorated ASD-induced CD in mice by modulating the NF-κB pathway and oxidative stress.

First, an ASD mouse model was established using the platform water environment method, which can simulate the mechanism of ASD caused by muscle relaxation under natural conditions, a mechanism that better mimics physiological ASD. In contrast, external interventions like electrical stimulation or gentle handling (e.g., cage tapping or novel object introduction) may introduce non-specific stress [50,51]. Moreover, the water environment method does not require continuous human intervention, thus reducing human interference with the natural behavioral patterns of mice [50]. The sleep–wake behavior was determined using EEG, and EEG results confirmed that SD mice remained awake for an average of approximately 95% of the time during the 18 h SD period over 3 consecutive days (Supplementary file 1). It has been documented that SD elevates path length and escape latency observed in mice to reach the hidden platform, as well as decreases time and activity range in the target area, and the number of traverses and time spent in the target area [52]. A study conducted by Cheng et al. has revealed that SD rats display sparsely arranged hippocampal CA1 neurons [53]. Consistent with previous studies, this study revealed that ASD could lead to CD in mice. Further, our findings unveiled the ameliorative role of melatonin in CD in ASD mice. In a similar light, melatonin facilitates sleep induction and coordinates the alignment of central and peripheral rhythms [54]. Meta-analysis showed that melatonin improved learning ability by reducing escape latency, and that it effectively corrected memory deficits by increasing the dwell time in the target quadrant and the number of crossings over the platform location [55]. Melatonin diminishes escape latency in rats in the MWM test on the third day following melatonin administration, and it attenuates chronic rapid eye movement SD-led hippocampal-dependent spatial memory and learning deficits [56]. Melatonin mitigates depressive behaviors and CD in mice through the regulation of the circadian rhythm of AQP4 polarization [57]. To conclude, melatonin relieved ASD-induced CD in mice.

SD has the potential to trigger neuroinflammation, a process that can subsequently facilitates the onset of oxidative stress, leading to eventual brain damage [5,6]. This study found that ASD caused increased levels of pro-inflammatory factors (TNF-α and IL-1β), reduced levels of anti-inflammatory factors (IL-4 and IL-10), elevated MDA and ROS levels, weakened SOD activity, and neuronal damage in the hippocampal CA1 region of mice, which were consistent with previous studies [58,59]. Melatonin demonstrates the capacity to decrease oxidative stress in the cerebral cortex and hippocampus [60]. The administration of melatonin to rats with permanent bilateral common carotid artery occlusion results in a notable decrease in neuronal cell damage and reductions in the levels of MDA, TNF-α, and IL-1β in the ischemic hippocampus [61]. Moreover, melatonin enhances PSD95 expression in hippocampal parvalbumin neurons [62]. Innovatively, this study manifested that melatonin attenuated neuroinflammation and oxidative stress in the hippocampal CA1 region of ASD mice.

SD activates the NF-κB pathway [63]. The upregulation of NF-κB has the potential to stimulate the expression levels of pro-inflammatory cytokine genes, which can consequently result in neuronal injury [17]. Furthermore, melatonin abrogates SD-caused increments in p-p65 and p-IκB proteins in the hippocampus and alleviates anxiety-like behaviors by improving oxidative stress, neuroinflammation, and NF-κB activation [64]. Melatonin also inverts SD-induced diminishment in IL-10 and increments of MDA, IL-6, TNF-α, p-IκB, and p-P65 proteins in the SD-induced small intestinal [65]. Intriguingly, this study revealed that melatonin inhibited the activation of the NF-κB pathway. A recent study proposed that inhibiting the activation of the NF-κB pathway is crucial for suppressing the inflammatory response [66]. Inhibition of the NF-κB pathway increases brain SOD activity, enhances PSD95, and reduces levels of TNF-α, IL-1β, NF-κB, and MDA in type 2 diabetes rats [67]. The inhibition of NF-κB suppresses crystalline silica-induced astrocyte activation and neuronal death in the mouse hippocampus [68]. Gastrodin improves rapid eye movement SD-induced cognitive deficits, sleep disturbance, and neuron damage in the hippocampus CA1 region by curbing the TLR4/NF-κB pathway and activating the Wnt/β-Catenin pathway [17]. Moreover, Zhang et al. have demonstrated that the NF-κB inhibitor down-regulates TNF-α, NF-κB, and IL-1β in rats treated with intermittent hypoxia [69]. Besides, the pretreatment of a NF-κB inhibitor improves hippocampal neuronal apoptosis and CD in rats exposed to chronic intermittent hypoxia [70]. PDTC restores NF-κB level to their control values, ameliorates cognitive deficits, and alleviates diabetes-induced behavioral dysfunction, as demonstrated by a decrease in latency to find the platform and an increase in the time spent in the target quadrant [71]. Innovatively, this study revealed that inhibition of the NF-κB signaling pathway ameliorated neuroinflammation and oxidative stress in the hippocampal CA1 region of ASD mice, as well as significantly attenuated CD in ASD mice.

Taken together, this study found that melatonin ameliorated ASD-induced CD in mice by modulating the NF-κB pathway and oxidative stress. Evidence has demonstrated the role of melatonin in reducing oxidative stress and inflammation in hippocampal damage models. However, this study is the first to analyze synaptic plasticity in ASD. We found that ASD led to a reduction in PSD95 expression [72], while melatonin could restore its expression. Moreover, a significant correlation exists between PSD95 restoration and cognitive function improvement. Although there are reports on melatonin’s inhibitory effect on NF-κB, there has been no direct evidence regarding NF-κB’s influence on ASD-induced oxidative stress, inflammation, and CD, or whether NF-κB serves as the key pathway for melatonin to mediate effects on these SD-induced dysfunctions [64,65]. This study discovered for the first time that PDTC plays a role in ASD-induced CD and that NF-κB activation partly reversed the beneficial effects of melatonin on ASD-induced oxidative stress, inflammation, and CD. These findings provide robust mechanistic evidence that NF-κB inhibition is central to melatonin’s effects. Notably, unlike previous single-dose studies [37,38,64], we are the first to compare the differential effects of two clinically relevant melatonin doses (20 vs 40 mg/kg) on synaptic plasticity (PSD95), inflammatory responses, oxidative stress, and CD. Additionally, in contrast to prior studies focusing on the whole hippocampus [60] or intestinal mucosa [65], we investigated NF-κB, inflammatory responses, and oxidative stress in the hippocampal CA1 region (the most vulnerable area in ASD). However, there are several limitations that need to be considered. First, this mechanism was not verified at clinical levels. Second, based on previous studies [22,29–31], male mice was selected in this study. We did not systematically assess the impact of sex differences on the effects of melatonin on ASD-induced oxidative stress and CD. Third, we did not further confirm the duration of melatonin’s effects in the ASD model. Fourth, following the methods of previous studies, we only measured the total distance traveled by mice in the open field experiment. Additionally, other behavioral experiments (such as the MWM and elevated plus maze test) were combined to jointly assess CD in mice [73–76]. The specific behaviors (rearing, grooming) of mice were not analyzed. Therefore, in the future, the clinical trials investigating the mechanism of melatonin on CD in ASD mice via the NF-κB pathway and oxidative stress shall be carried out. Simultaneously, we will include female mice for a comparative study to comprehensively analyze the impact of sex. Furthermore, we will optimize the experimental design by extending the observation period and establishing different time points to dynamically evaluate the time-dependent effects of melatonin intervention and its potential sustained effects. In addition, we will also fully consider and refine the content of behavioral assessments.

## Supplementary Material

Supplementary Figure
