# Supplementary material for: The role of melatonin in affecting cognitive dysfunction in acute sleep deprivation mice through the nuclear factor kappaB pathway and oxidative stress
Source: Transl Neurosci. 2025 Oct 7;16(1):20250379. doi: 10.1515/tnsci-2025-0379 (PMC12514685; doi:10.1515/tnsci-2025-0379)
Supplement: Supplementary Figure [file tnsci-2025-0379-sm.pdf]

Supplementary material

S1 Materials and methods

S1.1 Electrode implantation surgery

Under isoflurane anesthesia (4% for induction and 2% for maintenance), four stainless steel electroencephalogram (EEG) recording electrodes (XR-XE105, Softmaze, Shanghai, China) were placed epidurally over the frontal and parietal cortices. The electrode leads were attached to pins of a plastic integrated circuit connector that was subsequently secured in dental acrylic. Surgical incisions received a topical analgesic (4% lidocaine) and a triple antibiotic ointment (neomycin/polymyxin B/bacitracin). Mice underwent one week of post-surgical recovery before being linked to the recording equipment via a flexible tether for habituation.

S1.2 Determination of the sleep-wake behavior

EEG was employed to determine mouse sleep-wake behavior. Signals obtained from recording electrodes underwent initial amplification (100-fold gain) in the preamplifier unit of an electroencephalogram/electromyogram (EEG/EMG) monitoring system, coupled with initial high-pass filtering (first-order 0.5 Hz to eliminate low-frequency noise like DC offset). Signals were then relayed through the tether and a low-torque commutator to the EEG/EMG acquisition system. Within the amplifier conditioning unit, signals underwent additional amplification (50-fold gain), achieving a total amplification of 5000-fold (100 × 50). Furthermore, signals underwent additional high-pass filtering and 8th-order elliptic low-pass filtering. Signals were subsequently sampled at 500 Hz, with analog signals converted to digital format using a 14-bit analog-to-digital (A/D) converter. Finally, digitized signals were transmitted via USB to a personal computer-based acquisition and analysis software package for subsequent storage and analysis. Mouse sleep-wake behavior determination was performed by a trained and experienced experimenter blinded to animal identification and experimental conditions.

S2 Results

To confirm the establishment of the SD mouse model, we first assessed mouse sleep-wake behavior via EEG. The results revealed that SD mice remained awake for approximately 95% of the time during the 3-day, 18-h SD period (Figure S2, $P < 0.001$).

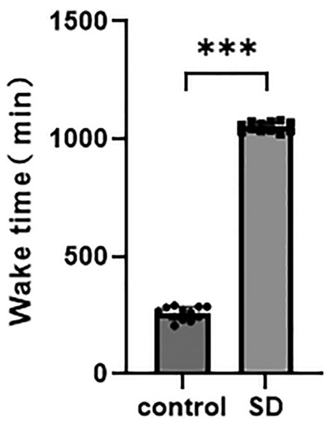

Figure S2: Wake time during the SD period in mice. EEG was used to record the wake time during the SD period in mice. $n = 12$. Data were presented as mean ± standard deviation. Comparisons between groups were performed using an independent sample t test. $^{***}P < 0.001$.

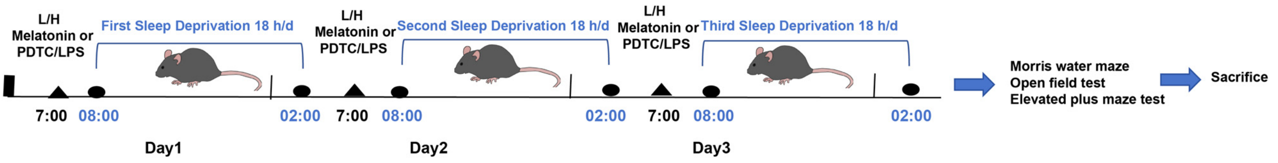

Figure S1: Experimental flow chart.
